# Role of T lymphocytes in tumor response to radiotherapy

**DOI:** 10.3389/fonc.2012.00095

**Published:** 2012-08-24

**Authors:** Sandra Demaria, Silvia C. Formenti

**Affiliations:** ^1^Department of Pathology, New York University School of Medicine and NYU Langone Medical CenterNew York, NY, USA; ^2^Department of Radiation Oncology, New York University School of Medicine and NYU Langone Medical CenterNew York, NY, USA

**Keywords:** abscopal, adjuvant, CD8 T cells, dendritic cells, immunoediting, immunotherapy, ionizing radiation, *in situ* vaccine

## Abstract

Over thirty years ago, Helen Stone and colleagues compared the effects of local tumor irradiation in immunocompetent and T cell deficient mice, providing the first evidence that tumor response to radiotherapy is impaired in the absence of a normal T cell repertoire. In the following three decades there has been an exponential growth in understanding T cells and the complex molecular mechanisms that regulate their activation, migration to tumors and effector functions. We now also know that tumor progression is intrinsically linked to the development of multiple immunosuppressive mechanisms that allow cancer cells to escape immune control. Recent evidence about the role of T cells in determining the prognosis and outcome of patients at any clinical stages of cancer has been instrumental in re-directing the concept of immunosurveillance and immunoediting from the realm of preclinical models to the reality of clinical observations. Importantly, cell death induced by standard anti-cancer therapies like chemotherapy and radiation has been demonstrated to involve the immune system and, in certain specific settings, enable a specific immune response. It is, therefore, not surprising that the last few years have seen an increase in investigations exploring how to harness the ability of radiation to induce anti-tumor immune responses. We will review here the experimental evidence that anti-tumor T cells are key players in tumor control achieved by radiotherapy. The effects of radiation on the tumor that have been shown to enhance the priming and effector phases of anti-tumor immunity will be discussed. Finally, we will highlight promising combinations of immune response modifiers that enhance T cell function with radiotherapy which are being tested in the clinic.

## Introduction

Ionizing radiation has been employed as a cancer treatment based on its cytocidal effects, and the response to radiotherapy linked mostly to the delivery of irreparable DNA damage to tumor cells. Therefore, research to improve the efficacy of radiotherapy has been dominated by studies of the mechanisms of DNA repair, their regulation in normal and neoplastic cells, and the tumor cell factors that affect radiosensitivity, such as the phase of the cell cycle. While *in vitro* these parameters are determinants of the inhibition of tumor cell growth by radiation, *in vivo* they are essential but not sufficient to explain the response of a tumor to local radiotherapy. In fact, a report published in 1979 by Helen Stone and colleagues demonstrated that *in vivo*, factors extrinsic to the cancer cell are key determinants of tumor radiosensitivity (Stone et al., 1979). Instead of studying the response of human tumor xenografts that grow only in immunocompromised mice, a mouse tumor was injected in syngeneic animals. Radiosensitivity was then compared in immunocompetent and T cell-deficient animals. The difference was striking: tumors growing in mice that lacked T cells required over 60 Gy to achieve the same tumor control obtained with 30 Gy in immunocompetent mice. More than thirty years later, the key role of T cells as anti-tumor effectors is unquestionable in experimental mouse models as well as in humans. There is evidence from clinical trials that adoptive transfer of tumor-specific T cells can eliminate tumors even at advanced stages (Porter et al., 2011; Restifo et al., 2012). Significant progress has also been made in understanding how a treatment considered immunosuppressive such as radiation can induce anti-tumor T cells, as reviewed in this article. While the clinical evidence of systemic anti-tumor responses from local radiotherapy is rare, the uncommon observation of tumor regression outside of the radiation field was recognized by R. H. Mole and named, in 1953 as abscopal effect from the latin “ab scopus,” i.e., away from the target (Mole, 1953). Based on the hypothesis that the abscopal effect is due to radiation-mediated induction of anti-tumor T cells (Demaria et al., 2004), interventions that improve T cell activation have shown abscopal effects when combined with radiotherapy in mice and humans (Demaria et al., 2005; Formenti and Demaria, 2009; Postow et al., 2012).

To understand the role of T lymphocytes in the tumor response to radiotherapy it is useful to review the evidence on the reciprocal influence that tumor and immune cells have on each other during tumor progression.

## Tumor-host immune system: a dynamic equilibrium

The fundamental task of the immune system is to maintain tissue homeostasis. This is an active process that requires a delicate balance between tolerance and active surveillance to detect any tissue change that is potentially dangerous. Since tissue turnover and physiological remodeling, for example in the breast post-weaning, are often associated with significant cell death, the immune system has developed sensors to distinguish it from pathogenic cell death. A key class of receptors devoted to triaging cell death are pattern recognition receptors (PRR). Expressed by innate immune cells they bind to pathogen-associated molecular pattern (PAMP) molecules derived from infectious agents and damage-associated molecular pattern (DAMP) molecules derived from cells dying a stressful death (Janeway and Medzhitov, 2002; Zeh and Lotze, 2005; Mills, 2011). The ability to resist cell death has been identified as one of the hallmarks of cancer (Hanahan and Weinberg, 2000), suggesting that, in addition to resulting in tumor growth, this property may also account for a failure of recognition of the pathogenic features of transformed cells by the immune system. However, there is plenty of evidence to the contrary and, in fact, immune recognition of cancer cells is so common that the ability to evade immune destruction has been increasingly recognized as an essential biological capability required by tumors in order to become clinically apparent (Hanahan and Weinberg, 2011). The cancer immunoediting theory provides a rationale for this apparent paradox (Dunn et al., 2002).

Neoplastic transformation is invariably associated with genomic instability and cell stress. Genomic instability leads to the generation of neoantigens-containing epitopes that can be recognized by T cells (Segal et al., 2008) and cell stress leads to the expression of molecules such as members of the family of NKG2D ligands that are recognized by natural killer (NK), γδ T cells and effector CD8 T cells (Diefenbach et al., 2000; Hayakawa et al., 2002). Local disruption of the stroma and of normal tissue architecture generates danger signals in the form of DAMPs, including degraded extracellular matrix components (e.g., heparin sulfate, hyaluronan) (Lotze et al., 2007) that attract innate immune cells. Recognition of the stressed neoplastic cells by NK or other innate immune cells results in production of interferon (IFN)-γ, a cytokine shown to play a key role in immunosurveillance against tumors (Street et al., 2001; Dunn et al., 2006). Killing of the neoplastic cells by NK cells or macrophages activated by IFN-γ to produce cytocidal reactive oxygen and nitrogen species, eventually leads to cross-presentation by dendritic cells (DC) of antigens from the dying tumor cells to T cells and activation of the adaptive immune system. Tumor-specific T cells may be able to completely destroy the incipient tumor, thus functioning as an extrinsic tumor suppressor mechanism that reduces the incidence of spontaneous and carcinogen-induced tumors. This is supported by unequivocal evidence in experimental models and indirect evidence in humans with various immunodeficiencies [reviewed in Dunn et al. (2004) and Vesely et al. (2011)]. However, if complete elimination of genomically unstable cells is not achieved, the immunological pressure results in selection of clones of neoplastic cells that have acquired, via mutations or epigenetic changes, resistance to immune rejection, i.e., are “edited” by the immune system to become poorly immunogenic. This transition from elimination to escape can occur directly, or sometimes can occur after a long period of equilibrium, during which the immune response is able to prevent or limit the progression of cancer. The concept of equilibrium, initially formulated to explain clinical observations of occult tumors and tumor dormancy (Myron Kauffman et al., 2002; MacKie et al., 2003), has been confirmed in experimental models: depletion of T cells leads to growth of occult tumors that are more immunogenic, indicating that much of the immunoediting occurs during the equilibrium phase (Koebel et al., 2007). Importantly, recent evidence demonstrates that CD8 T cells play a key role in “editing out” strongly immunogenic tumor antigens (DuPage et al., 2012; Matsushita et al., 2012).

The same property that allows tumors to escape immune control may become their Achille's Heel. Tumors with high levels of genomic instability due to microsatellite instability (MSI) are prone to generate novel tumor antigens. They are often highly infiltrated by T cells and their carriers often enjoy better clinical outcomes, an association suggestive of better immune control (Buckowitz et al., 2005; Chiaravalli et al., 2006). Importantly, the association between infiltration by CD8 T cells and improved prognosis is not exclusive to tumors with MSI (Zhang et al., 2003; Galon et al., 2006; Pagès et al., 2010). This observation suggests that the degree and type of immune response matters at every stage of tumor progression, including metastatic disease. For example, the ability of immunotherapeutic strategies to improve survival of patients with metastatic melanoma (Hodi et al., 2010) indicates that even in advanced stages, when tumors have escaped immune control, it is possible to enhance anti-tumor T cell reactivity to revert to a phase of equilibrium, even in the presence of more extensive tumor burden.

Tumor's escape from immune control is a complex process, which does not only occur via antigenic loss. To avoid immune rejection tumors exploit multiple pathways that physiologically maintain immune tolerance to “self” and protect healthy tissues from immune destruction during acute inflammatory reactions. The recruitment of suppressive, tolerogenic and regulatory innate and adaptive immune cells, the secretion of immune suppressive cytokines and the induction of dysfunctional differentiation of T cells can be seen in most, if not all tumors [reviewed in Demaria (2012)]. In addition, cancer cells downregulate major histocompatibility complex (MHC) class I molecules that are required for recognition by CD8 T cells (Chang and Ferrone, 2007), and upregulate immunosuppressive receptors that preclude their destruction by T cells (Dong et al., 2002). The tumor vasculature also presents multiple barriers to T cell infiltration, through an abnormal architecture and a relative paucity of endothelial adhesion molecules (Chen et al., 2003). Overall, the tumor microenvironment evolves into a protective hub for the neoplastic cells, that actively prevents tumor rejection. In this context, ionizing radiation acts as a modifier of the microenvironment with the potential to switch the immunosuppressive hub into an immunogenic one (Demaria and Formenti, 2007).

## Role of the immune system in response to local radiotherapy

Although radiation has been known to have pro-inflammatory and immunomodulatory effects for a long time (McBride et al., 2004), it is only recently that some of these changes have been elucidated at a molecular level. These studies have provided evidence for the counterintuitive concept that local radiotherapy, rather than suppressing anti-tumor immunity, can promote it. A series of important findings in relation to the main barriers to immune rejection that are affected by radiation have emerged.

As mentioned above, the correct assessment of cell death by innate immune cells as “dangerous” or “non-dangerous” dictates which downstream pathways are triggered to either activate adaptive immunity or maintain tolerance. The traditional dichotomy of cell death as apoptotic and non-inflammatory versus necrotic and inflammatory has been challenged by the demonstration that apoptotic death can be associated with release of pro-inflammatory and danger signals (Galluzzi et al., 2007). The stressful death of cancer cells induced by some types of chemotherapy and by ionizing radiation can be quite immunogenic and promote the cross-presentation of tumor-derived antigens by DC to T cells, leading to development of anti-tumor responses (Ma et al., 2010; Zitvogel et al., 2010). Among the three molecular signals identified as critical for the successful induction of immunogenic cell death, both, translocation of calreticulin (CRT) to the surface of the dying cell and release of high-mobility group protein B1 (HMGB1), which binds to the PRR Toll-Like Receptor (TLR) 4, are induced by ionizing radiation (Apetoh et al., 2007; Obeid et al., 2007). The third signal, active release of ATP by cells committed to apoptotic death, which is required to activate the NLRP3 inflammasome (Ghiringhelli et al., 2009) is still awaiting confirmation in irradiated cells. Given recent evidence that autophagy is required for ATP release (Michaud et al., 2011), and that ionizing radiation promotes autophagy (Rieber and Rieber, 2008; Rodriguez-Rocha et al., 2011), this third signal is likely to be generated by radiotherapy when autophagy precedes cell death. Overall, experimental evidence supports the contention that radiation can induce a tumor cell death that is perceived by the immune system as dangerous and, therefore, generates an *in situ* cancer vaccine.

**Figure 1:**
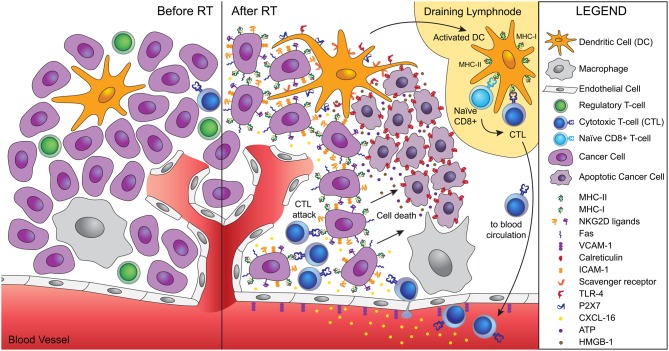
**Ionizing radiation acts as a modifier of the tumor microenvironment converting the tumor into an *in situ* vaccine.** Radiation induces an immunogenic cell death of tumor cells characterized by calreticulin translocation to the surface of dying cells, and release of HMGB-1 and ATP. Calreticulin allows uptake of dying cells by dendritic cells via scavenger receptor(s). HMGB-1 binds to TLR4 and promotes the cross-presentation of tumor antigens, while ATP binds to P2X7 and triggers the activation of the inflammasome. Activated dendritic cells migrate to the draining lymph node, where they activate naïve T cells specific for tumor antigens. Activated CD8 T cells acquire effector functions and traffic to the tumor guided by radiation-induced chemokines. Tumor infiltration by CTLs is facilitated by radiation-induced upregulation of VCAM-1 on the vascular endothelium. Once in the tumor, CTLs interact efficiently with tumor cells expressing increased levels of MHC-I, ICAM-1, NKG2D ligands, and Fas that promote the formation of stable immunological synapses between targets and effectors and facilitate the killing of tumor cells by CTLs. Tumor cells killed by CTLs become a source of antigens for cross-presentation, thus fueling the process.

Once activated, T cells have to be able to home to and infiltrate the tumor. Radiation has been shown to promote this process in multiple ways. For instance, radiation-induced remodeling of the abnormal tumor vessels, resulted in efficient tumor infiltration by adoptively transferred anti-tumor T cells in a spontaneous mouse tumor model (Ganss et al., 2002). In a murine experimental model of melanoma, up-regulation of vascular cell adhesion molecule (VCAM)-1 induced by radiation increased infiltration by T cells, in a process requiring IFN-γ production (Lugade et al., 2005, 2008). Our group demonstrated in a poorly immunogenic mouse carcinoma that radiation-induced up-regulation of the chemokine CXCL16 was required for the efficient recruitment to the tumor of CXCR6^+^ effector CD8 T cells, resulting in optimal tumor inhibition (Matsumura et al., 2008). Other important effects of radiation include the up-regulation of MHC class I molecules, adhesion molecules, NKG2D ligands, and Fas/CD95, enhancing the ability of effector T cells to bind to and kill the cancer cells (Hareyama et al., 1991; Gaugler et al., 1997; Chakraborty et al., 2003, 2004; Garnett et al., 2004; Gasser et al., 2005; Kim et al., 2006; Newcomb et al., 2006; Reits et al., 2006). Thus, radiation is a significant modifier of tumor microenvironment with specific effects that facilitate tumor rejection (Figure 1).

Despite the multiple pro-immunogenic effects, radiation by itself is usually insufficient to generate strong and lasting T cell responses that in addition to contributing to eradicate the irradiated tumor can control the growth of established metastases. Multiple immunosuppressive pathways make it very difficult to overcome these barriers by radiotherapy alone, in the absence of additional interventions. However, addition of antibodies to block a negative regulator of T cell activation, the checkpoint receptor cytotoxic T-lymphocyte-associated antigen 4 (CTLA-4), induced therapeutically significant anti-tumor immunity to a poorly immunogenic carcinoma treated with local radiotherapy, while each treatment by itself was not effective (Demaria et al., 2005). In addition, radiation induces effects that can dampen the immune response, like the activation of transforming growth factor (TGF)β (Jobling et al., 2006), and a relative increase in regulatory T cells (Kachikwu et al., 2011). Altogether, the pre-existing balance between tolerogenic and effector anti-tumor mediators, and the degree to which radiation can induce activation without stimulating suppression, converge to determine the outcome in terms of local and systemic tumor control. Intriguingly, there is at least some evidence that the type of the radiation regimen employed may have a role in determining whether a favorable pro-immunogenic response is elicited (Dewan et al., 2009).

## Harnessing the pro-immunogenic effects of radiation in cancer treatment: a new paradigm

Progress in understanding the function and dysfunction of the immune system in cancer has identified specific targets for intervention, based on the dominant immunosuppressive mechanism in a given tumor type and/or patient (Zitvogel et al., 2011). The growing evidence that local radiotherapy can generate an *in situ* vaccine supports its use in concert with personalized immunotherapy, since the killed tumor cells provide the entire antigenic diversity of a patient's own tumor.

Since DC function is often suboptimal in tumors, studies have tested strategies to increase DC numbers and function by administering DC growth factors in combination with radiotherapy. Experimental work in two syngeneic mouse models, a lung and a mammary carcinoma, employed Flt-3 ligand as growth factor to expand DC, and demonstrated the induction of a T cell-mediated response that reduced tumor growth outside the field of radiation (Chakravarty et al., 1999; Demaria et al., 2004). Based on this data, we conducted a clinical trial that used s.c. GM-CSF to increase the percentage of DC and their maturation and facilitate cross-presentation of newly released antigens, after cell death at the site of radiotherapy. We selected patients with at least 3 metastatic sites from solid tumors. With a standard radiation fractionation of 3.5 Gy X10 fractions delivered to one tumor site we were able to measure an out-of field (abscopal) response in 30% of the patients with metastatic solid tumors accrued to the trial (Formenti and Demaria, 2009). In murine models, exogenously prepared DC injected in the tumor following radiation induced anti-tumor immune responses (Nikitina and Gabrilovich, 2001; Teitz-Tennenbaum et al., 2003; Kim et al., 2004). These effects were translated in the majority of patients with hepatoma and high risk sarcoma treated in two early clinical trials (Chi et al., 2005; Finkelstein et al., 2012). In preclinical models molecular mimics of the danger signals associated with pathogens, like olygodeoxynucleotides containing CpG motifs that bind to TLR9, when injected intratumorally enhanced DC activation and ability to cross-present tumor antigens released by radiation (Milas et al., 2004; Mason et al., 2005). A similar combination of local radiotherapy and CpG administration was tested in 15 patients with low-grade B-cell lymphoma, showing abscopal responses, associated with development of tumor-specific T cells (Brody et al., 2010). Taken together, the data support the ability of radiation to generate an *in situ* vaccine: the efficacy of this approach is dependent on DC fitness and can be enhanced by interventions directed at improving DC.

A complementary strategy is based on targeting checkpoint co-inhibitory receptors or co-stimulatory receptors expressed by T cells with blocking or agonistic antibodies, respectively, to achieve stronger and more sustained responses of anti-tumor T cells. Our group tested the hypothesis that inhibiting a key checkpoint receptor, CTLA-4, in combination with radiotherapy would induce therapeutically effective anti-tumor responses. While CTLA-4 is a dominant inhibitory receptor for T cells, as demonstrated by the development of uncontrolled T cell proliferation in mice deficient in CTLA-4 (Chambers et al., 1997), CTLA-4 blockade as monotherapy failed to induce regression of poorly immunogenic tumors, requiring its use in combination with vaccination (Peggs et al., 2008). Therefore, we hypothesized that radiotherapy would synergize with anti-CTLA-4, due to its ability to generate an *in situ* vaccine. This hypothesis was confirmed in mice models of poorly immunogenic carcinomas (Demaria et al., 2005; Dewan et al., 2009). The therapeutic efficacy of the anti-tumor T cells activated by treatment was enhanced by other effects of radiation such as an improved tumor infiltration by effector T cells, confirming it's beneficial effects at both the priming and effector phase of anti-tumor responses (Matsumura et al., 2008). A recent case report suggests that the success of the combination of local radiotherapy and anti-CTLA-4 can be translated in melanoma patients (Postow et al., 2012), with multiple clinical trials being conducted to confirm these results.

Targeting of other co-stimulatory or co-inhibitory receptors expressed by T cells, CD137 and programmed death (PD)-1, respectively, has also shown some success in combination with radiation in mice models (Newcomb et al., 2010; Verbrugge et al., 2012), supporting more studies to develop these strategies for clinical use.

A number of other studies exploited the pro-immunogenic effects of local radiotherapy that promote the effector phase of tumor rejection, by combining radiation with either vaccination or adoptive immune therapy (AIT). Increased expression of MHC class I antigens by irradiated glioma cells was implicated in the synergy of peripheral vaccination with whole brain radiation (Newcomb et al., 2006). In a mouse carcinoma, radiation-induced Fas expression was shown to synergize with T cell AIT and with vaccination, by facilitating tumor cell killing by T cells (Chakraborty et al., 2003, 2004). Interestingly, following the combination of vaccine and local radiation there was an induction of T cells specific for tumor antigens not present in the vaccine, a phenomenon known as antigen cascade or antigenic spread. Similarly, antigen cascade was also observed in prostate cancer patients treated with standard definitive radiotherapy and vaccination (Gulley et al., 2005).

## Conclusions

Immune response modifiers (IRM) have been defined by the National Cancer Institute Translational Research Working Group as “immunotherapy agents that mimic, augment, or require participation of the host immune system for optimal effectiveness” (Cheever et al., 2008). Although host T cells contribution to the optimal tumor response to radiation was demonstrated over three decades ago (Stone et al., 1979), it is only in the last decade that the underlying mechanisms begun to be understood. Increasing number of publications testing new combinations of radiation and immunotherapy testify to the growing interest toward a new role of radiation as an “immunological adjuvant”. Most exciting is the emerging evidence that radiation may indeed function as an IRM in patients, suggesting that it may be time to consider a paradigm shift in the use of radiotherapy.

### Conflict of interest statement

The authors declare that the research was conducted in the absence of any commercial or financial relationships that could be construed as a potential conflict of interest.
